# Use of the specific binding ratio distribution to characterise multiple system atrophy in advanced iodine-123-labelled N-(3-fluoropropyl)-2β-carbomethoxy-3β-(4-iodophenyl) nortropane serotonin transporter imaging

**DOI:** 10.22038/aojnmb.2024.78274.1557

**Published:** 2025

**Authors:** Kazuya Takahashi, Masanobu Ishiguro, Yoshitaka Inui, Takashi Ichihara, Cong Shang, Ryunosuke Nagao, Yasuaki Mizutani, Mizuki Ito, Hirohisa Watanabe, Nobutoku Motomura, Hiroshi Toyama

**Affiliations:** 1 Department of Radiology, Fujita Health University School of Medicine, Aichi, Japan; 2 Section of Radiology, Fujita Health University Hospital, Aichi, Japan; 3 Department of Neurology, Fujita Health University School of Medicine, Aichi, Japan; 4 Canon Medical Systems, Otawara, Tochigi, Japan; † Kazuya Takahashi and Masanobu Ishiguro contributed equally to this study.

**Keywords:** Serotonin transporter, SPECT, Binding potential, Multiple system atrophy

## Abstract

**Objective(s):**

Sudden death in multiple system atrophy (MSA) is caused by decreased serotonergic innervation, but there is no routine test method for this decrease. In addition to dopamine transporters, iodine-123-labelled N-(3-fluoropropyl)-2β-carbomethoxy-3β-(4-iodophenyl) nortropane (^123^I-FP-CIT) binds serotonin transporters (SERTs). We noted a binding potential to quantify the total quantity of ^123^I-FP-CIT binding to its receptors.

Following Mintun’s binding-potential concept, this study aimed to evaluate the relationship between the specific binding ratio (SBR) and total SERT tissue amount, but not SERT binding, and to develop an SBR imaging method to measure brain-stem SERT. We sought to establish a binding-potential imaging procedure using SBR images to examine differences in the brain-stem SERT distribution between healthy subjects and MSA patients.

**Methods:**

Single-photon emission computed tomography (SPECT) and T1-weighted magnetic resonance (MR) images were aligned. The MR (T1) images were used to set a reference site for the occipital-lobe SBR in each subject, and measurements were made from the SPECT image at the same position. The pixel values and accumulation ratios compared with the occipital lobe were calculated, and a regional SBR distribution image was created. We identified areas with SERT accumulation above a certain level.

**Results:**

The SERT accumulation site was visualised as an SBR value on MR images. The accumulation distribution (SERT distribution) on the SBR images significantly differed between the healthy subjects and patients with MSA.

**Conclusion:**

SERT accumulation was noted in the brain-stem region, indicating that SBR imaging was useful for viewing and quantifying SERT accumulation.

## Introduction

 The compound iodine-123-labelled N-(3-fluoropropyl)-2β-carbomethoxy-3β-(4- iodophenyl) nortropane (^123^I-FP-CIT) has high affinity for the dopamine transporter (DAT). Therefore, ^123^I-FP-CIT is a useful tool for assessing the status of presynaptic nigrostriatal dopaminergic terminals and is widely used in the diagnosis of Parkinson’s disease (PD) and parkinsonian syndrome. ^123^I-FP-CIT also exhibits a significant affinity for the serotonin transporter (SERT) (2, 3). This compound is a proven effective tool for in vivo examination of SERT regions given that the binding ratios within the SERT-abundant midbrain, thalamus and hypothalamus are conspicuously attenuated by administration of citalopram, a selective serotonin reuptake inhibitor (4). Positron emission tomography (PET) studies using [^11^C]-3-amino-4-(2-dimethylaminomethyl-phenylsulfanyl)-benzo-nitrile (DASB) have revealed that modifications in the extrastriatal serotonergic system are significantly correlated with clinical mani-festations of PD (5-7). Furthermore, three studies previously used Statistical Parametric Mapping and the PETPVE12 toolbox to analyse the specific binding ratio (SBR) to investigate the binding of ^123^I-FP-CIT single-photon emission computed tomography (SPECT) to SERT outside the striatum in PD (8-10), and all three studies generated SBR from the SPECT image and used it as an index of the amount of binding between ^123^I-FP-CIT and SERT. However, the spatial resolution, sensitivity and attenuation-correction (AC) methods of SPECT were different.

 In contrast, in cerebral SPECT examinations, the study of ligand accumulation in cortical areas with low SERT is considered unreliable, as shown in a previous study (4). Furthermore, because head shape and skull density vary per individual, nonuniform-AC using CT, among other factors, is reportedly essential for quantifying cerebral blood flow and DAT imaging in brain SPECT (11, 12). On the basis of these studies, we performed CT-AC in a multicentre ^123^I-iodoamphetamine-SPECT evaluation and unified the spatial resolution to reduce the variability of SPECT values between institutions and improve quantification (13).

 The present study aimed to evaluate the relationship between the SBR value and SERT total amount, but not SERT binding, present in tissues, on the basis of the binding-potential concept proposed by Mintun (1). The total amount of SBRT can be considered as the number of free specific binding sites, and the SBR value is proportional to the total amount of SBRT. We also sought to determine if it is possible to observe differences in SERT distribution in the brain-stem region by comparing SBR images between healthy subjects and patients suffering from multiple system atrophy (MSA), a disease known to cause widespread serotoninergic system involvement, as determined in pathological studies (14-17).

## Methods

***Participants***

 The study involved 17 healthy subjects (mean±SD; 72±9.2 years old, 8 males, 9 females) and 11 patients with MSA (60±9.2 years old, 5 males, 6 females) who underwent head MRI and DAT SPECT (including head CT for CT-AC) in the ‘Multicentre joint study targeting healthy adult volunteers for collecting ^123^I- FP-CIT-SPECT healthy adult data (medical research ethical review: receipt number HM16-254)’ and the description of MSA-related applications. All patients with MSA fulfilled the diagnostic criteria for probable MSA (18). We focused on MSA-P because of the pronounced atrophy of the brainstem observed in MSA-C, which could confound the interpretation of our results. By initially focusing on MSA-P, we aimed to mitigate the potential impacts of structural atrophy on serotonin transporter distribution.

***Equilibrium model***

 In this study, we adopted an equilibrium analysis using a three-compartment model (1) to analyse the intake of ^123^I-FP-CIT (‘radioligand’) into the striatum and midbrain. 

**Figure 1:**
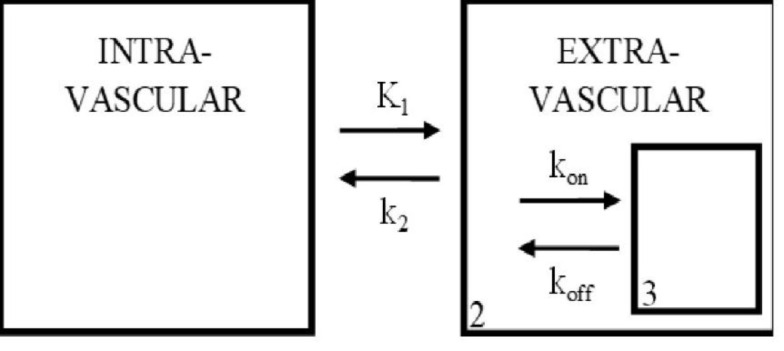
Three-compartment model used in analysis of the brain tissue. Compartments 1, 2 and 3 represent the possible environments for the radioligand. Interactions between compartments are governed by variables for diffusion, and binding kinetics (k_on_, k_off_). This figure is a modified version of the one reported by Mintun et.al (1984) (1)

 The schematics and symbols of this model are shown in Figure 1 and Table 1, respectively. The first two compartments are physical compartments that represent blood and brain tissue. The third compartment represents the chemical binding conditions of a specific binding site. Changes in time per unit volume in compartment 3 are represented by the following set of differential equations (see the Appendix for details):

$\frac{dC_{3}}{\mathrm{dt}}=\frac{1}{V_{2}}\left\{k_{\mathrm{on}}{f_{2}C}_{2}\left(B_{\max}-C_{3}\right)V_{2}\right\}-\frac{1}{V_{2}}k_{\mathrm{off}}C_{3}V_{2}\cdots (1)$

 Considering the equilibrium state 3 h after injection (3), the relationships between B_max _and rate constants were expressed by setting the derivatives to zero in Eq. 1. In the DAT SPECT examination, when the specific activity of the radioligand is assumed to be sufficiently high and the molar quantity of the injected ligand is relatively negligible, the absolute value of C_3_ is far lower than that of B. Therefore, (B_max_ − C_3_) can be reduced to (B_max_), and the following equation (2) is derived:

Bmaxkoffkon=C3f2C2⋯(2)

 Where K_D_=k_off_/k_on_, C_3_ / (f_2_C_2_)=B_max_/K_D_, is in proportion to B_max_, and K_D_ remains relatively unchanged in the population. B_max_/K_D_ is described as the ‘binding potential’ (BP). From the derivation of this theoretical formula, this BP reflects the capacity of the tissue for ligand-binding-site interactions (1). 

 Assuming that no dopamine or SERTs are in the occipital region, Eq. 2 can be expressed as follows (19):

Bmaxkoffkon=C3f2C2

 Which is approximately equal to [(cpm (count per minute) / pixel) _brain stem_ − (cpm / pixel) occipital)]/ (cpm/pixel) occipital⋯(2A)

**Table 1:** Variables Used in the Drug Binding Model

**Symbol**	**Description**	**Units**
K_1_	Forward rate constant	ml・sec^−1^・μCi^−1^
k_2_	Reverse rate constant	sec^−1^
V_i_	Volume of compartment i	ml
C_i_	Drug concentration in compartment i	μCi ml^−1^
f_i_	Drug fraction free from nonspecific binding	None
k_off_	Reverse rate constant	sec^−1^
k_on_	Forward rate constant	ml・sec^−1^・μCi^−1^
B_max_	Maximum drug specific binding concentration	μCi ml^−1^
BP	Binding potential of specific binding	None

***Data acquisition***

 First, the consent of each volunteer was confirmed, and medical interviews and head MRI examinations were conducted. Based on the interview results and the radiographical interpretation of the head MRI results, the suitability of the subject was confirmed, and DAT SPECT using ^123^I-FP-CIT was performed later. ^123^I-FP-CIT (Ioflupane (^123^I)) 167 MBq (radioactivity level on the test date) was administered, and SPECT images were acquired 3 h after injection.

 SPECT imaging was performed using a triple-head SPECT system (GCA-9300R, Canon Medical Systems) (20). To acquire SPECT data, a FANHR collimator in the SPECT continuous rotation mode, 3 detectors ×120 degrees/ detector, 90 projection (4 degrees/ projection), 6 min/rotation ×5 rotations was used, with a total acquisition time of 30 min. For the projection data, TEW (Triple Energy Window) scattered correction (21) (filter: no main-window, lower Sub-window Butterworth 

[Order 4, 0.25 cycles/cm]) was implemented. 

 From the fan-beam projection data count of 90 (matrix: 128×128, 4 degrees/step), rebinning was performed to the parallel-beam projection data count of 60 (matrix: 128×128, 6 degrees/ step). For the projection data, prefiltering (Butterworth filter, order 8, cutoff of 0.58 cycles/cm) was implemented, and 3D ordered subset expectation maximisation (OS-EM) (iteration 10, subset 10), including spatial resolution correction, was used to reconstruct the data. 

 Concurrently, using the GCA-9300R's built-in application, CT images were imported and aligned with SPECT images, and then CT-AC was performed. SPECT images were obtained along all three axes at a resolution of 7–8 mm full width at half maximum (FWHM) (22).

 The MRI system used was a Titan 3-T MRI (Canon Medical), and the CT apparatus was a SOMATOM Definition AS_mCT. Scan data were acquired according to the protocol (described in Table 2).

**Table 2:** Imaging parameters of ^123^I-FP-CIT SPECT, head MRI, and CT

**SPECT imaging parameters**		
Energy window	Main	159 kev±10%
	Sub	Main±7 %
Matrix		128×128
Pixel size		1.72 mm
Sampling angle		4°
Sampling mode		Continuous
SPECT sampling time		30 min
**CT imaging parameters**		
Tube voltage	120 kV	
Tube current	Automatic tube current modulator	
Matrix	512×512	
Slice thickness	2.00 mm	
Pitch	1.50 mm	
**MRI imaging parameters**		
MRI imaging sequence	3D-T1-WI	
Matrix	256×256	
Slice thickness	2.00 mm	
TR/TE	7.2 msec/3.4sec	

***SBR image generation and analysis***

**Figure 2:**
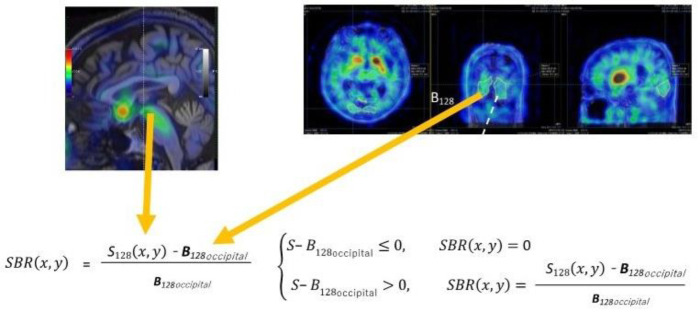
Specific binding ratio (SBR) to non-SBRs imaging. The above formula SBR(x, y) is calculated for whole-brain regions. At this time, if the SBR value is negative, it will be replaced with 0, and if it is positive, it will be that value. S_128_ represents the single-photon emission computed tomography (SPECT) value in the striatum and brain stem, and B_128_ represents the SPECT value in the occipital-lobe region with no specific binding to dopamine transporter and SERTs. When measuring B_128_, a region of interest (ROI) is set to three-dimensionally enclose the entire occipital-lobe region, and within that ROI, voxel-wise averages are calculated for imaging

 MIRADA DBx ver.1.1.1 (Mirada Medical) was used to align the T1-weighted MR and SPECT images. First, to measure the reference value, the MR (T1) images were used to set the 3D region of interests (ROIs) in the occipital region of the SPECT image. To minimise the variation in reference values, the 3D ROI was set largely over the entire occipital lobe (the lower limit of the threshold was set at 50% of the max SPECT value per the threshold method), and the means within the ROI were measured and used as reference values in the reference value site. The reason for setting the occipital as the reference region is that 1) it is an area that is not easily affected by MSA, and 2) it is also commonly used as a reference in PD. Next, Eq. 2A was used to subtract the reference value of the occipital lobe from the SPECT pixel value (^123^I-FP-CIT accumulation) per SPECT image pixel, and the ratio relative to the reference site of the occipital lobe was calculated and Vitria (Canon Medical Systems) was used to create an SBR image (formulas 3 and 4 below) (Figure 2).

$S_{128}\left(x,y\right)-B_{128}\leq 0,\mathrm{SBR}\left(x,y\right)=0$ (3) 

$S_{128}\left(x,y\right)-B_{128}>0,\mathrm{SBR}\left(x,y\right)=\frac{S_{128}\left(x,y\right)-B_{128}}{B_{128}\left(x,y\right)}$ (4)

 Where $S_{128}\left(x,y\right)$ in counts/voxel is the SPECT value of the pixel at position (x, y) and $B_{128}$ in counts/voxel in the cortex of the occipital lobe=(nonspecific binding and free radioactivity).

***Visual evaluation of the accumulation sites***

**Figure 3:**
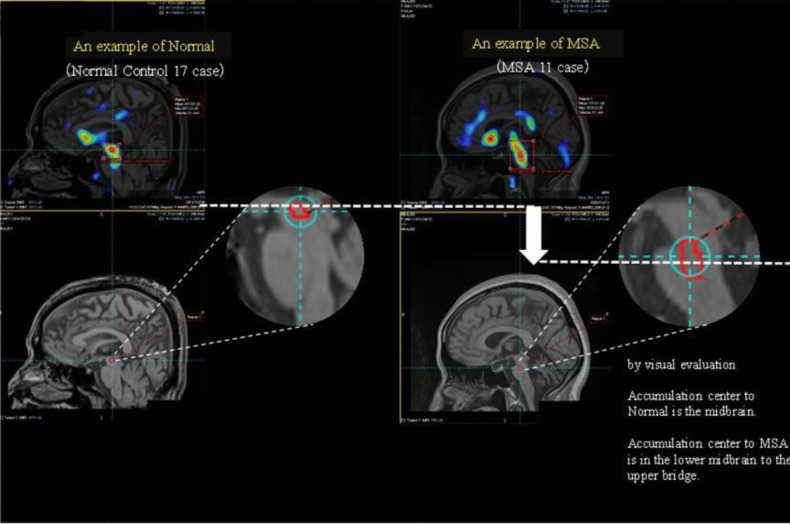
Examples of typical accumulation distribution in the Normal and in patients with multiple system atrophy (MSA). In the visual evaluation, the accumulation centre is the midbrain in Normal, and it is from the lower midbrain to the upper pons in MSA

 Mirada XD software was used to perform multimodality image registration of the MRI and SBR images and to evaluate the SBR images (Figure 2). An accumulation area (3D-ROI) was created by setting a threshold of 0.8 in the SBR image on the MRI image so that the maximum value and the position in that area were identified. The threshold value was set at 0.8 because the maximum value of the brain stem area in the SBR images of healthy subjects was often around 1.6, so it was easy to identify the range of distribution by setting the ROI within one half of that maximum. They were then considered as the area and centre of accumulation, respectively (Figure 3).

**Figure 4:**
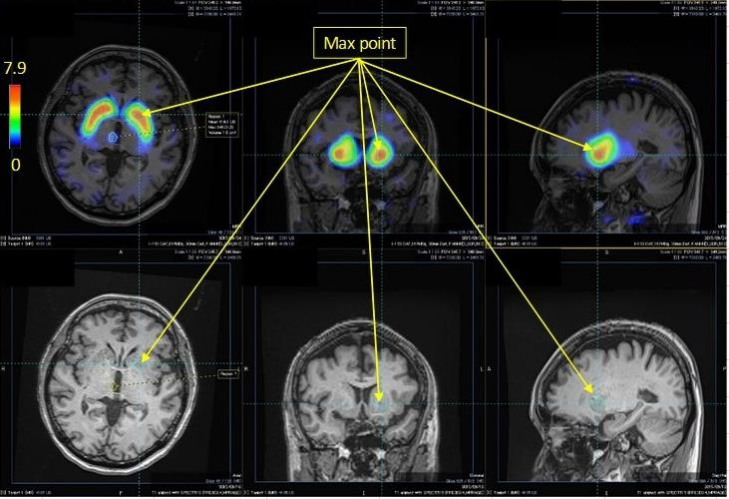
An example of a specific binding ratio (SBR) image of the striatum. DAT Dopamine transporter accumulation is localised in the striatum, as revealed by overlapping MRI the magnetic resonance image, and the maximum SBR value is 7.9

**Figure 5:**
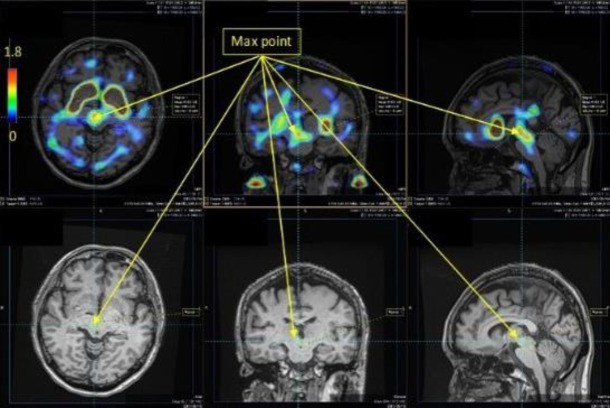
An example of a specific binding ratio (SBR) image of the brain stem. Serotonin transporter accumulation is localised in the brain stem, as revealed by overlapping the magnetic resonance image, and the maximum SBR value is 1.8. This is lower than the SBR maximum value in the striatum

 Three radiologists (K.T., S.S., H.T.) visually reviewed these images (Figures 4, 5) and identified the set accumulation areas. The results were compared.

## Results

Figures 4 and 5 show the SBR images of the striatum and brain stem, respectively. Figure3 shows the typical SERT distribution patterns in the healthy subjects and patients with MSA, and for each accumulation pattern, the number of subjects is listed in Table 3. As shown in Figures 3 and 5, the serotonin transporter-accumulated sites were visualised on MRI images as SBR values. Visual evaluation revealed that the centre of SERT accumulation was the midbrain in normal patients and that it varied from the lower midbrain to the upper pons in the patients with MSA (Table 3). Among the healthy subjects, 13 were in the upper midbrain, 4 in. the lower midbrain, and 0 in. the pons. Among the patients with MSA, 3 were in the upper midbrain, 6 in the lower midbrain and 2 in the pons. When the accumulation in the brain stem of the healthy subjects and patients with MSA (Table 3) was analysed by use of Fisher’s exact test in R version 4.3.1, the P value was 0.013 (<0.05), indicating a significant difference.

**Table 3:** Visual evaluation results

**Subject**	**Accumulation center**	**Number of subjects**
Nomal	Upper midbrain	13
	Lower midbrain	4
	Pons	0
MSA	Upper midbrain	3
	Lower midbrain	6
	Pons	2

## Discussion

***SBR images are BP distribution images***

 Bolt et al. calculated and quantitatively evaluated DAT accumulation in the striatum as an SBR value per unit volume (accumulation concentration) (23). Although SBR images were produced in this study, the significance of the values remained unclear. SBR is the ratio of DAT accumulation in an accumulation area to free DAT, and its significance was not well discussed in Bolt’s report. Thus, we tentatively preferred to base our study on Mintun’s report (1), which was the first to propose a BP, and perhaps on later reports. However, in the later reports, we found that the definitions and meanings of model variables and parameters were ambiguous, including whether or not the movement of tracers was caused by diffusion or chemical reaction. Therefore, in the present study, we returned to the original model proposed by Mintun et al. (1) and established a new method to generate SBR images. 

 Consequently, we confirmed that the binding potentials equalled the SBR images. This finding means that the total quantity of BP in cerebral tissue [i.e. receptor (in this case, the number of SERT binding sites) × (affinity of the ligand for binding to the SERT binding site)] and SERT distribution images can be directly extrapolated from SPECT images by use of ^123^I-FP-CIT, which is a radioligand. Notably, we need to emphasise that an SBR image does not provide a distribution image of the radioligand ^123^I-FP-CIT bound to the site but instead shows a distribution image of ‘the number of SERT-binding sites present in the tissue’. In the present study, SBR represented BP itself. 

 Therefore, comparing BP in the entire striatum between patients may help clinicians detect differences in pathological conditions.

***Differences in the SERT accumulation regions***

 Hashimoto et al. (12) reported that the results of SBR measurement using ^123^I-β-CIT varied greatly depending on the ROI. To improve the accuracy and reproducibility of the resultant SBR values, we performed SPECT imaging with TEW scatter and AC using CT images and reconstructed 3D OS-EM images to improve resolution. A possible SERT accumulation area was consequently observed in the brain-stem region, which indicated that BP distribution images tended to show differences between normal and MSA samples.

***Relationship between SBR distribution and serotonin levels***

 Our study showed considerable disparities in the SBR distribution between patients with MSA and healthy subjects. Given that the SBR distribution image can be interpreted as analogous to the binding-potential image, we postulate that it represents individual variations in receptor distribution. Patients with MSA showed a reduction in serotonin neurons in the medulla oblongata and in 5-hydroxyindoleacetic acid levels, which is the principal metabolite of serotonin in the spinal fluid (14, 24). Consequently, it can be inferred that SBR distribution alterations in patients with MSA may correlate with serotonergic neuronal cell attrition. Serotonin deficiency could contribute to respiratory disorders and sudden death (15-17). A more recent PET study with DASB showed an inverse correlation of SERT abundance with the severity of the Movement Disorders Society Unified Parkinson’s Disease Rating Scale motor score in patients with MSA (7). Further studies are needed to elucidate the relationships between SERT SPECT SBR image findings, clinical indices, and pathological findings. 

***Limitations***

 There were some study limitations that should be considered when interpreting the results. It is crucial to note that the loss of serotonin neurons does not necessarily correlate with changes in serotonin transporter levels, a phenomenon observed in other neurological disorders. For example, studies on autism have shown an increase in serotonin axons in the brain (25), suggesting that alterations in the serotonin system can be influenced by disease-specific mechanisms that do not directly lead to neuronal loss. Therefore, further detailed examination of postmortem cases is necessary to fully understand the specific changes in serotonin transporters in MSA. This deeper analysis could lead to a better understanding of the pathophysiology of the serotonergic system and aid in the development of targeted therapeutic strategies. We plan to incorporate more detailed postmortem data in our future studies.　Further pathological examination is essential to address these questions. In addition, we plan to incorporate quantitative analyses in future research to provide a more detailed and rigorous evaluation of these changes. By doing so, we aimed to better understand the pathological underpinnings and potentially divergent patterns of serotonin transporter dynamics in MSA. We plan to include patients with MSA-C in future studies to broaden our understanding and confirm whether the patterns observed are consistent across different subtypes of MSA.

## Conclusion

 Our results indicate that SBR images may be useful in identifying and quantitatively analysing SERT accumulation areas in neurodegenerative diseases, such as MSA.

## Appendix

The radioligand migrates away from the blood compartment via venous drainage at a concentration equal to that in the blood compartment. The radioligand enters the tissue compartment through the blood–brain barrier via passive linear diffusion. It then freely reacts with the drug-binding site only within the tissue compartment. Moreover, nonspecific, unsaturated radioligand-binding sites are present within the blood and tissue compartments. The quantity of drugs that freely diffuse or react with the binding site is represented as a value calculated by multiplying the total drug concentration by the free-fraction constant (f_i_). The volume of the radioligand entering - i.e. reacting with the binding site - the chemical environment compartment is represented as a value multiplied by the following three factors:

(k_on_: forward rate constant) × (concentration of free radioligand within brain tissue) × (number of free specific binding sites). Herein, the number of free specific binding sites is represented as (B_max_: maximum drug-specific binding concentration) − (C_3_: drug concentration in compartment 3).

The differences in the total inflow and outflow into each compartment represent the changes in time in each compartment. Changes in time per unit volume in compartments 2 and 3 are represented by the following set of differential equations divided by the compartmental volume:

$\frac{dC_{2}}{\mathrm{dt}}=\frac{1}{V_{2}}\left\{K_{1}C_{A}+k_{\mathrm{off}}C_{3}V_{2}\right\}-\frac{1}{V_{2}}\left\{k_{2}f_{2}C_{2}+k_{\mathrm{on}}{f_{2}C}_{2}\left(B_{\max}-C_{3}\right)V_{2}\right\}$

$\frac{dC_{3}}{\mathrm{dt}}=\frac{1}{V_{2}}\left\{k_{\mathrm{on}}{f_{2}C}_{2}\left(B_{\max}-C_{3}\right)V_{2}\right\}-\frac{1}{V_{2}}k_{\mathrm{off}}C_{3}V_{2}$

Koopman et al. reported that specific/ nonspecific ^123^I-FP-CIT binding ratios in the midbrain and diencephalon remained stable for 2–3 h after injection (3). Therefore, considering the equilibrium state 3 h after injection, the relationship between B_max_ and rate constants was expressed by setting the derivatives to zero in Eq. 1.

$0=k_{\mathrm{on}}{f_{2}C}_{2}\left(B_{\max}-C_{3}\right)-k_{\mathrm{off}}C_{3}$

Since the absolute value of C_3_ is much smaller than B, as shown above, (B_max_ − C_3_) in the above equation can be reduced to (B_max_), which can be transformed into the following equation (1):
